# Polydimethylsiloxane (PDMS) Membrane for Separation of Soluble Toluene by Pervaporation Process

**DOI:** 10.3390/membranes13030289

**Published:** 2023-02-28

**Authors:** Salam H. Rasheed, Salah S. Ibrahim, Qusay F. Alsalhy, Hasan Sh. Majdi

**Affiliations:** 1 Membrane Technology Research Unit, Department of Chemical Engineering, University of Technology-Iraq, Alsinaa Street 52, Baghdad 10066, Iraq; 2 Department of Chemical Engineering and Petroleum Industries, Al-Mustaqbal University College, Babylon 51001, Iraq

**Keywords:** pervaporation, PDMS, toluene, design of experiment, response surface methodology

## Abstract

A commercial polydimethylsiloxane (PDMS) membrane was employed to separate the soluble toluene compounds (C_7_H_8_) from an aqueous solution via the pervaporation (PV) process. The performance and the efficacy of the PDMS PV membrane were evaluated through the estimation of the permeation flux and separation factor under various operating parameters. The response surface method (RSM) built in the Minitab-18 software was used for the design of the experiment in this study, and the responses of the permeation flux and the separation factor were analyzed and optimized based on the operating conditions. A nonlinear regression analysis was applied to the experimental output and input, and as a result, a quadratic equation model with parameters interactions was obtained as mathematical expressions to predict the permeation flux and separation factor. At the optimal conditions of temperature 30 °C, initial toluene concentration 500 ppm, and feed flowrate 3.5 L/min, the toluene permeation flux and separation factor were 125.855 g/m^2^·h and 1080, respectively. The feed concentration was the most impactful and significant in the improvement of the permeation flux and separation factor of the PDMS membrane.

## 1. Introduction

The environmental damage brought on by our economic activity has, in recent decades, become a problem. Because of their potentially detrimental effects on the ecosystem and the human body, both directly and indirectly, volatile organic compounds (VOCs) are a particular concern for water pollution. Therefore, it is highly desired to create technology to remove diluted VOCs from water [1].

Nowadays, numerous industries, including biotechnology, food, the chemical industry, wastewater purification, and desalination, use membrane filtering methods widely, in part because of their low energy requirements. To separate liquid mixtures, separation techniques have been developed in recent decades. One such technique is the PV process. This method combines membrane permeation and evaporation to separate liquid molecule combinations in a specific manner [2]. In contrast to the PV process, other separation processes, such as the Azeo/extractive distillation technique, liquid–liquid extraction, and drying agents technique, do not require any additional chemicals and operate at low temperatures and ambient feed pressure. Further, PV is an energy-saving process with low operating, maintenance, and capital costs. Moreover, it is an environmentally friendly and pollution-free technique (green separation technique); however, PV processes require purified feed, and temperature reduction in pervaporation reduces the transmembrane flux [3]. A dense selective layer of an asymmetric membrane separates a liquid mixture through the PV membrane process. Extensive testing of PV is being performed on systems that are challenging for current separation techniques, such as distillation, adsorption, and extraction. As a matter of fact, PV is a strong contender for the separation of azeotropic and close-boiling liquids, heat-sensitive materials, and organic mixtures, as well as the removal of diluted volatile organic compounds (VOCs) from wastewater and the recovery of volatile aroma compounds from fruit juices [4,5,6,7,8,9,10].

Given that the world population is predicted to grow by 40–50% by 2050, there is a growing interest in providing water that is suitable for human use [11]. However, the proper disposal of VOCs must be performed by using more up-to-date, efficient, and affordable techniques in order to preserve the environment and aquatic life from pollution. Many industrial applications now use organic solvents in their technological processes to produce refrigerants, plastics, adhesives, paints, petroleum products, and so on [12,13,14,15,16,17]. As a result of these various industrial activities, large amounts of VOC-polluted water will be dumped into rivers, endangering both humans and the environment [18,19,20]. VOC solubility in water is typically very low, implying that concentrations of these substances in water are low and, at the same time, hazardous to the environment. Fortunately, the PV method can effectively treat VOCs because it is sufficient for doing so without the use of expensive separation techniques, including distillation, oxidation, biological treatment, and adsorption [21]. Many researchers have studied the PV process to treat VOCs-polluted water under a variety of operating conditions and membranes. Khayet et al. [22] used a commercial PDMS membrane to remove acetonitrile from the aqueous solution by the PV process. Zhou et al. [23] also developed PV mixed matrix membranes (MMMs) based on silicalite-1 and PDMS for ethanol separation from aqueous solutions. Cai et al. [24] studied the removal of ethanol, acetone, and butanol from fermentation broth by using a polydimethylsiloxane/polyvinylidene fluoride (PDMS/PVDF) membrane. Lazarova et al. [25] used a poly(octhylmethyl siloxane) (POMS) membrane to recover ethanol from fermentation broth. Jian et al. [26,27] prepared poly (vinylidene fluoride)—(PVDF flat) and (PVDF hollow fiber) to separate the benzene compound from water.

Of the VOCs, toluene (C_7_H_8_, the pollutant under study) is a clear and colorless liquid. The chemical formula of toluene can be written as C_6_H_5_CH_3_ because it has a methyl group. It is used for paints, glues, printing ink, and leather tanners [28].

The International Agency for Research on Cancer IARC (1990) concluded that there is inadequate evidence for the carcinogenicity of toluene in both experimental animals and humans and classified it into Group 3 (not classifiable as to its carcinogenicity to humans). Nevertheless, the predominant effects were impairment of the central nervous system and irritation of mucous membranes [29]. Therefore, researchers are trying to find solutions to remove these substances. Among the various methods of water treatment, the PV process is one of the most promising ways to remove organic pollutants. Hamouni et al. [2] used polydimethylsiloxan (PDMS) to separate toluene from water at a temperature of 40 °C, a pressure of 9–10 mbar, a concentration of 5–25 wt %, and an active area of 46.55 cm^2^. The flux was 0.675–0.786 kg/m^2^·h., and the separation factor 29–24. Matavos et al. [30] removed toluene from water by using pure polyether-block-amide (PEBA) membranes with different thicknesses (25, 50, and 75 μm) and PEBA/2 wt % NaX nanozeolite at a temperature of 25 °C, a pressure of 1 kPa, a concentration of 50–400 ppm, and an active area of 24.6 cm^2^. Salehi Shahrabi et al. [31] used polydimethylsiloxan (PDMS) + polyethersulfune (PES) as a composite membrane with a temperature of 30–50 °C, a pressure of 1 mbar; the concentration of toluene was 150–300 ppm and the active area was 10 cm^2^. The flux was 0.0035–0.0075 kg/m^2^·h, and the separation factor was 1300–2200. Panek and Konieczny [32] employed polyether-block amide (PEBA) membrane and polyether-blockamide (PEBA)+ carbon black to separate toluene from aqueous solution with a temperature of 25 °C, a pressure of 100 Pa, a concentration of 500 ppm of toluene, a flowrate of 1.77 L/min, and an active area of 100 cm^2^. For polyether-block amide (PEBA) membrane, the flux was 0.0132 kg/m^2^·h, and the separation factor was 1500. For polyether-blockamide (PEBA)+ carbon black, the flux was 0.01614 (kg/m^2^·h), and the separation factor was 900. Panek and Konieczny [33] used PDMS with carbon block (cb) with the same conditions above, where the flux was 0.01732 kg/m^2^·h, and the separation factor was 300; using (PDMS) only, the flux was 0.01827 kg/m^2^·h, and the separation factor was 160. Nijhuis et al. [34] used a polydimethylsiloxan (PDMS) membrane to separate toluene–water solution, and they noticed that the flux of water decreased with the increase in the thickness of the membrane, where the flux of water changed from 0.051 to 0.006 kg/m^2^·h, whereas the flux of toluene changed from 0.020 to 0.013 kg/m^2^·h—it became clear by practical experience that the flux of toluene is affected to a lesser extent than the flux of water when the thickness of the membrane increases. On the other hand, by using ethylene propylene rubber and polyoctenamer membrane (EPDM), the flux of water changed from 0.0009 to 0.00017 kg/m^2^·h, and the flux of toluene changed from 0.0085 to 0.0035 kg/m^2^·h.

Diverse hydrocarbon molecules, including ethanol [35], acetone and acetonitrile [36], and butanol [37], were separated from water using a commercially available PDMS membrane made by DeltaMem AG (Switzerland) for the pervaporation process. However, no research utilizing developed PDMS membranes to separate soluble toluene from water has yet to be published in the literature. Therefore, the current study concentrated on the PV process and a PDMS membrane’s ability to separate the soluble toluene component from an aqueous solution under various operating conditions. Polydimethylsiloxane (PDMS) is the most common material for the preparation of hydrophobic membranes because its structure containing a siloxane (Si-O) backbone substituted with methyl groups guarantees high chemical stability and a highly hydrophobic character. PDMS is a very flexible polymer because of the lack of double bonds that allows a high degree of rotation of the bonds, facilitating the diffusion of permeating species through the free volume; the glass temperature of PDMS is 150 K (−123 °C), and as a result, PDMS-based membranes often have a higher flux for organics than glass-state membranes. PERVAP™ 4060 is a composite membrane consisting of a very thin separation layer on top of a porous support coated on a mechanical support (polymer fleece). To avoid a significant change in the initial toluene concentration in the feed, a large volume of the feed solution was used, and the amount of permeate was kept below 0.5% of the initial feed load [38]. The response surface method (RSM) was used for the experiment design, and the responses of the permeation flux and the separation factor were analyzed and optimized based on the operating conditions. Moreover, mathematical models were developed in this study that connects the significant variables with the anticipated responses.

## 2. Materials and Methods

### 2.1. Materials

The commercial hydrophobic membrane (PDMS Pervap™ 4060) used in this research was supplied by DeltaMem AG, Allschwil, Switzerland. The toluene (99.5% purity) was purchased from Lab-scan, Ltd., Dublin, Ireland. Distilled water was used to prepare all of the aqueous solutions. All characteristics and properties of the PDMS Pervap™ 4060 membrane were presented in our previous work [38].

The PDMS membrane consists of three layers: the first one is the active layer with a thickness of 5.5 µm, the second layer is the support layer with a thickness of 77.5 µm, and the third layer is the nonwoven fabric layer with a thickness of 101.5 µm. Therefore, the total thickness of the membrane is 184.5 µm. 

### 2.2. Pervaporation Process

**Figure 1:**
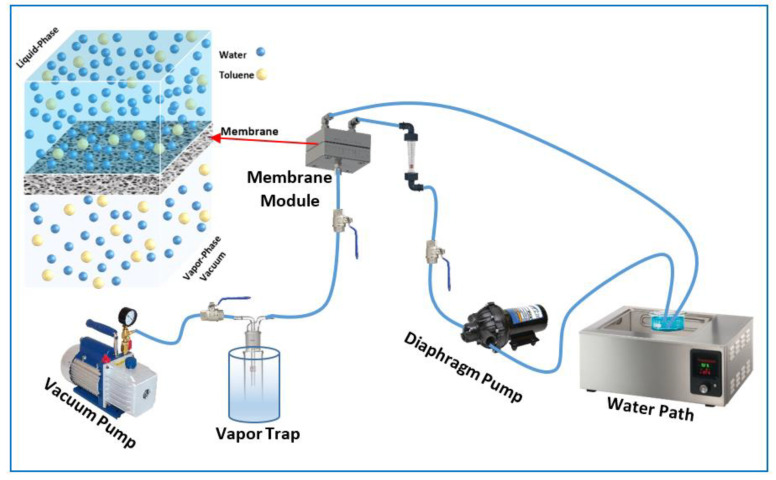
Schematic diagram of the pervaporation process.

The lab-scale setup used for the tests on the pervaporation process is shown schematically in Figure 1. A mixture of toluene–water with a volume of 1500 mL was initially used as a feed solution. A thermal digital water bath (DK-8AXX, MEDITECH, Taichung, China) was used to maintain the mixture feed temperature at a range of 30 to 50 °C. A diaphragm pump (BD, 400GPD, Waterpal International Co., Ltd., Kaohsiung City, Taiwan) was used to pump the feed mixture to the membrane cell. The concentration of toluene in the feed solution was in the range of 100 to 500 ppm, and the feed flowrate was from 1.5 to 3.5 L/min. The PDMS membrane, with an effective area of 26.5 cm^2^, was supported by a perforated plate. A single-stage vacuum pump (B-42, Sigma, Shanghai, China) maintained the vacuum pressure downstream of the module at 2.0 kPa. Permeate samples were gathered in a vapor trap submerged in liquid nitrogen. A computerized balance (SARTORIUS AC, Goettingen, Germany) was used to weigh the permeate with a 0.001 g precision. The content of toluene and water in the permeate was measured using a UV-visible spectrophotometer (V-630, Jasco, location, max = 260 nm, Tokyo, Japan).

The permeation flux (*J*) and separation factor (*S.F.*) can be used to evaluate the performance of the membrane in the PV process. The separation factor, which consists of two materials (*i* and *j*), such as toluene–water, is defined as the ratio of the mole fraction of the components in the permeate to that in the feed. The permeation flux is the rate of transporting the targeted substance through a unit area of a membrane during a given time.

The results of permeation flux and separation factor were estimated by using Equations (1) and (2):

(1) $$J=\frac{w}{A\times t}$$

(2) $$S.F.=\frac{y_{i}/y_{j}}{x_{i}/x_{j}}$$

where w is the weight of the permeate; t is the experimental time; A is the effective area of the membrane; $y_{i}$, $y_{j}$, $x_{i}$, and $x_{j}$ are the mole fractions in the permeate (y) and the feed (x) in relation to the toluene and water, respectively.

### 2.3. Experimental Design

Ease of dealing with statistical programs, as it saves a lot of time and effort that you can spend to obtain the mathematical model that links the different variables with each other, in addition to the possibility of obtaining the best results in optimal operating conditions. The double effect of the variables can also be illustrated through the graphics that will be obtained using these programs. In this work, the response surface method (RSM), built with Minitab 18 software, was used in contrast to prior studies that used a range of techniques to create comparable experiments (such as the Taguchi method) [39,40]. RSM is a collection of mathematical and statistical methods for developing, enhancing, and optimizing processes. It may be used to evaluate the relative importance of various aspects, even when there are intricate relationships [41]. To ascertain the link between the elements impacting the output in this work, the system required 20 experimental runs in which all factors were adjusted simultaneously during a series of tests. The best operating conditions and the ideal reaction were also found using the experimental design program [22,42].

## 3. Results and Discussion

### 3.1. Feed Temperature

**Figure 2:**
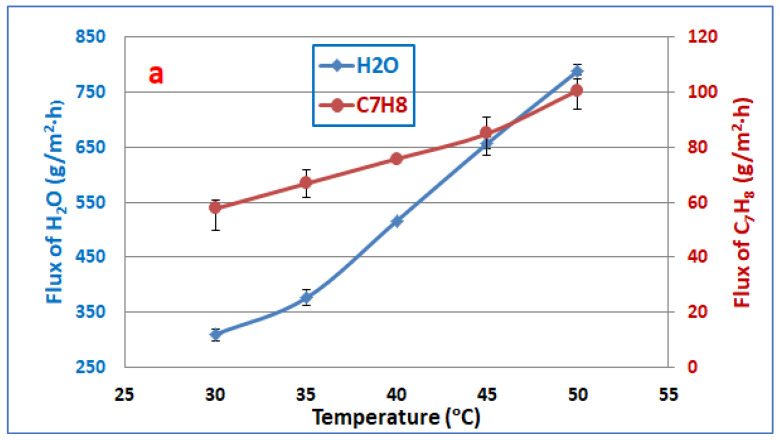

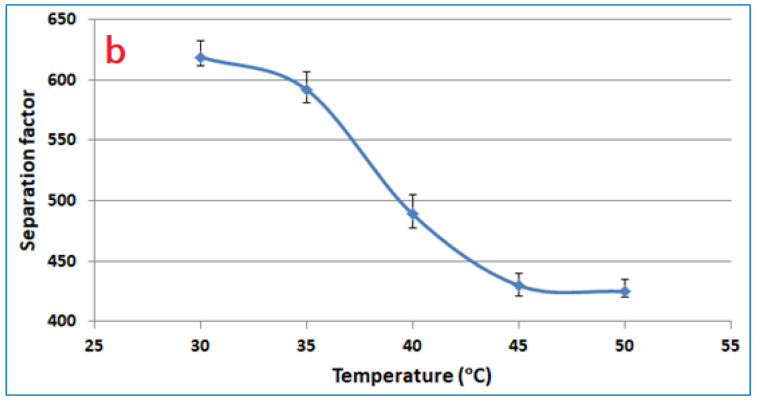
Effect the temperature on (**a**) fluxes of toluene and water, (**b**) separation factor, at toluene feed concentration of 300 ppm and 3 L/min feed flowrate.

Figure 2a illustrates the impact of toluene–water feed temperature on the permeation flux of toluene and water at a toluene feed concentration of 300 ppm and 3 L/min feed flowrate. According to the manufacturer’s instructions, the maximum long-term operating temperature is 80 °C for PERVAP™ 4060; therefore, the experiments were carried out between 30–50 °C to protect the membrane from damage. It can be seen that the toluene flux increased from 57.7 to 100.75 g/m^2^·h, whereas the water flux increased from 310.9 to 789.8 g/m^2^·h as the temperature of the feed mixture increased from 30 to 50 °C. These results were due to an increase in the distance between polymer chains, which resulted in an increase in the free volume of molecular transit. Moreover, as the temperature increases, the component’s vapor pressure increases, resulting in increased driving force across the membrane, which in turn, increases the permeation flux of all components [43]. Moreover, as seen in Figure 2b, an increase in feed temperature increases the toluene and water permeation fluxes. In this case, the increase in the permeation flux of water is higher than the permeation flux of toluene. Generally, in the PV process, the diffusion of the permeating molecules is carried out across the free volumes of the membrane. The increment in the free volume of the membrane with increasing temperature is due to the movement of the polymer chains. Therefore, with large free volumes, the membrane can have a lower separation factor, as depicted in Figure 2b. This behavior was already found in the literature [44,45].

### 3.2. Feed Concentration

**Figure 3:**
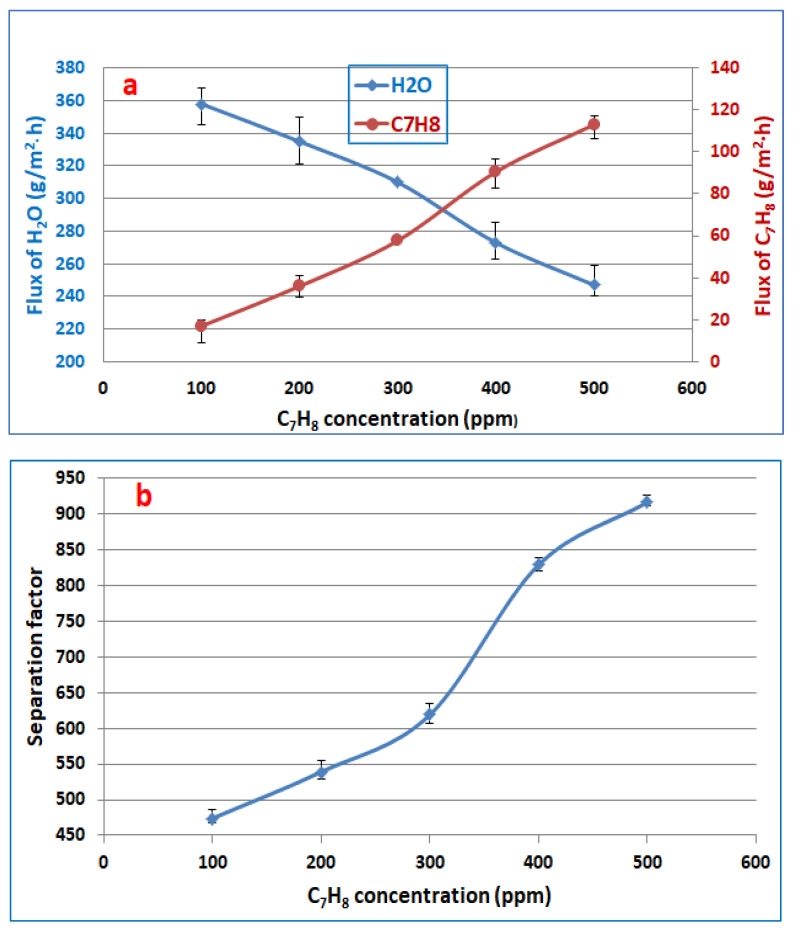
Effect the toluene feed concentration on (**a**) fluxes of toluene and water, (**b**) separation factor, at feed temperature of 30 °C and 3 L/min feed flowrate.

The concentration of toluene used in this work was from 100 to 500 ppm because the toluene component’s solubility in water can be 520 ppm at a temperature of 20 °C [28]. Figure 3 shows the effect of toluene feed concentration on fluxes of toluene and water as well as the separation factor at a feed temperature of 30 °C and 3 L/min feed flowrate. It can be noticed that the permeation flux of toluene increases from 16.98 to 113.2 g/m^2^·h with increasing concentrations of toluene in the feed from 100 to 500 ppm, as depicted in Figure 3a. The driving force between the upstream and downstream pressures across the membrane increases with increasing toluene concentration in the feed [31]. The water flux was decreased from 357.8 to 247.2 g/m^2^·h with an increase in the concentration of toluene in the feed solution, as depicted in Figure 3a. This result may be explained by the fact that the water molecules clustered due to the hydrogen bonding between water molecules, which in turn reduces their diffusivity and permeability. In Figure 3b, the separation factor increased from 474 to 915 as the toluene content in the feed solution increased from 100 to 500 ppm. Water clustering is developed in the membrane as a result of the repellent reaction between water and toluene, and it has been conclusively shown that the development of a water cluster may impede the passage of water through polymer membranes [45].

### 3.3. Effect of Feed Flowrate on the Toluene Partial Flux

**Figure 4:**
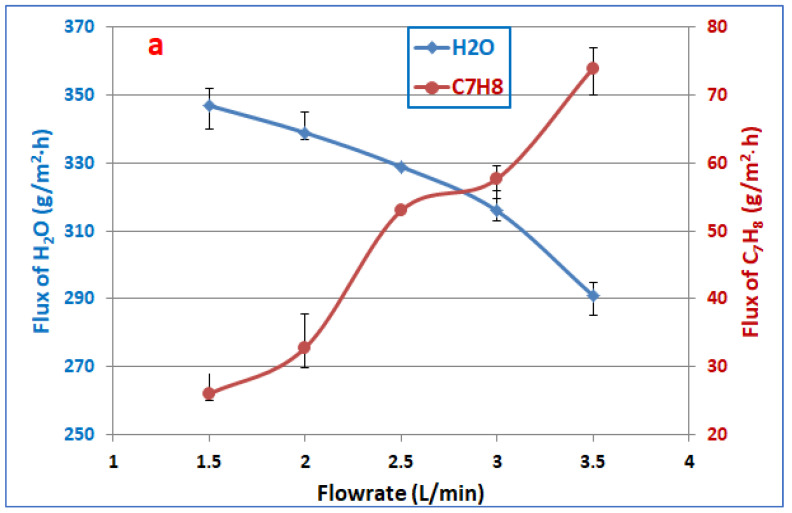

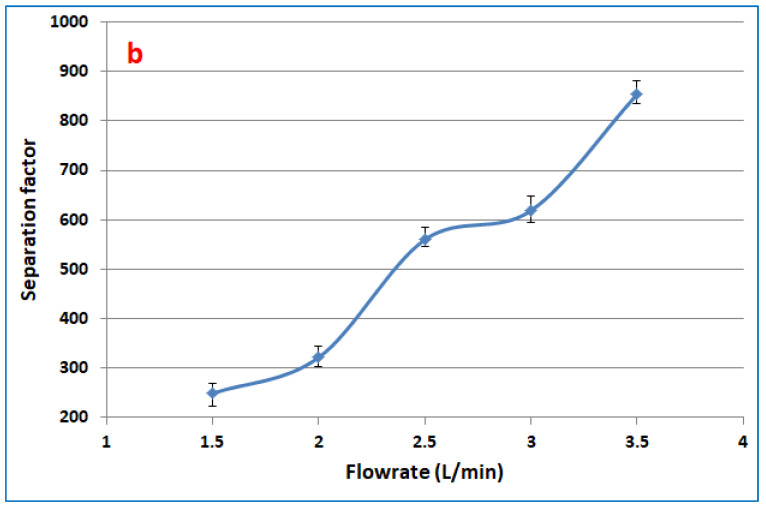
Effect the feed flowrate on (**a**) fluxes of toluene and water, (**b**) separation factor, at 300 ppm toluene feed concentration and 30 °C feed temperature.

Figure 4 shows the effect of the feed flowrate on fluxes of toluene and water and the separation factor of the PDMS membrane at 300 ppm toluene feed concentration and 30 °C feed temperature. It can be noticed that the toluene partial flux improved from 26 to 74 g/m^2^·h with an increase in the feed from 1.5 to 3.5 L/min. It was observed that the effect of the flow rate on the flux of toluene was strong, may be attributed this observation to reduce the concentration polarization effect on toluene permeation in pervaporation after increasing the flow rate, besides the minor thickness of the active layer, which was about 5 µm. The boundary layer represents a major resistance for toluene transport in addition to the permeation resistance in the membrane, where the flux was inversely proportional to the active layer thickness of the membrane [46,47]. As a matter of fact, the boundary layer effect is significant, as the flow regime is close to laminar (i.e., Re = 1117–2607, for the range of feed–solution velocity investigated). Concentration polarization tends to decrease the permeation rate of a more permeable component (which in this case is toluene) and increase the permeation rate of the less permeable component (i.e., water in this case), resulting in a lesser extent of separation; however, an increase in the feed–solution velocity could reduce the effect of concentration polarization, and thus the toluene flux should increase as observed; this agrees with that reported in the literature [44,45]. The effect of concentration polarization might be diminished by increasing feed flowrate, and this would also result in a thinner boundary layer. As a result, there was less resistance to the material passing through the membrane, while the flux of water decreased from 347.4 to 291 g/m^2^·h. Figure 4b illustrates that the separation factor increases with an increase in the flowrate of the feed due to the increasing permeation flux of toluene and decreasing water flux; the following equation is a good indication of this phenomenon [48].

(3) S.F.=ciperm.cjperm.cifeedcjfeed=JiJjcifeedcjfeed

where $c_{i}\mathrm{perm}.$ and $c_{i}\mathrm{feed}$ are the concentration of the compound *i* in the permeate and feed, respectively; $c_{j}\mathrm{perm}.$ and $c_{j}\mathrm{feed}$ are the concentration of the compound *j* in the permeate and feed, respectively; and $J_{i}$ and $J_{j}$ are the permeate flux of the compounds *i* and *j*, respectively.

### 3.4. RSM

#### 3.4.1. Predicted Model and ANOVA Calculations

The Minitab 18.1 software program was used to determine the operating parameters for the RSM experimental data points, and Table 1 presents the experimental outcomes that indicated the responses of the permeate flux and separation factor of the PDMS PV process.

Mathematical formulas to forecast the responses of the PV process were developed using the examination of the results of toluene permeate flux and separation factor. The equations for the toluene permeate flux and separation factor were obtained using Minitab 18 software, and the following quadratic nonlinear regression model was proposed:

(4) $$J_{T}=A_{0}+A_{1}\;T+A_{2}\;C+A_{3}\;F+{\;A}_{4}{\;T}^{2}+A_{5}C^{2}+A_{6}{\;F}^{2}+A_{7}\;T\;C+{\;A}_{8}T\;F\;+{\;A}_{9}\;C\;F$$

(5) $$S.F.=A_{0}+A_{1}\;T+A_{2}\;C+A_{3}\;F+{\;A}_{4}{\;T}^{2}+A_{5}C^{2}+A_{6}{\;F}^{2}+A_{7}\;T\;C+{\;A}_{8}T\;F\;+{\;A}_{9}\;C\;F$$

where the coefficients from A_0_ to A_9_ are illustrated in Table 2. 

**Figure 5:**
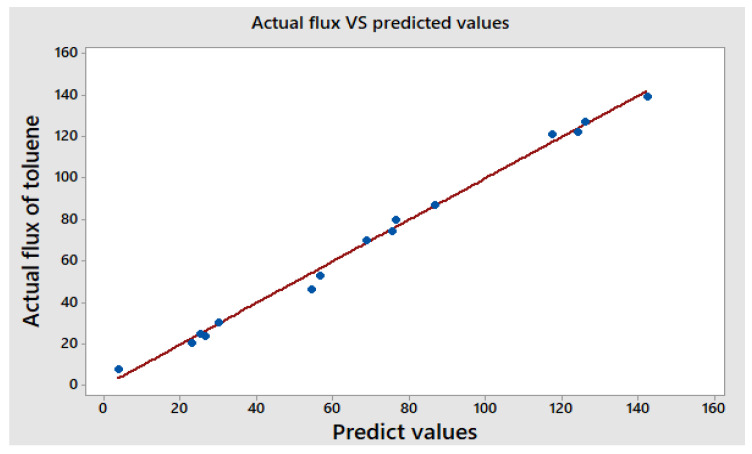

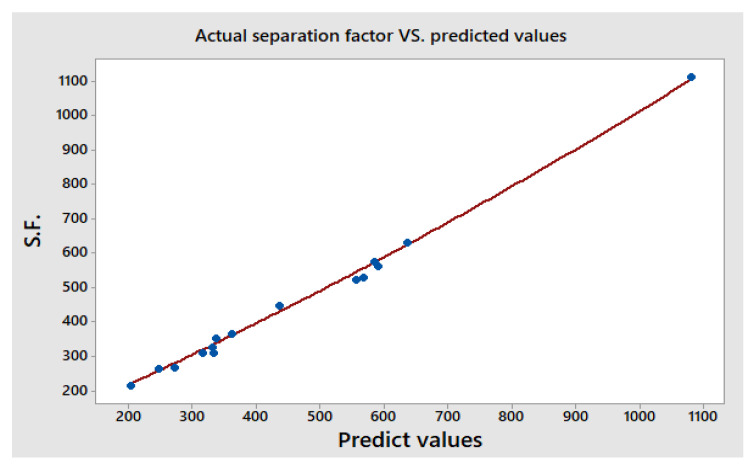
The relationship between actual and predicted toluene flux and separation factor (*S.F.*).

Figure 5 compares the experimental data from the trials listed in Table 1 with the values of the toluene permeate flux and separation factor estimated from Equations (4) and (5). Figure 5 shows a good agreement between the experimental findings and the suggested models (regression formulae).

Using Minitab 18 software, the analysis of variance (ANOVA) was calculated to properly quantify the importance of each element. It is useful in finding the impact of different factors. Moreover, the ANOVA test helps determine the significance or non-significance of the results of an experiment. It is possible to calculate the sum of square (SS), which is equal to Σ(*x*−*y*)^2^, where (*x*) is equal to the sum of measurement data divided by the number of experiments and (*y*) is measurement data. The mean square is equal to (SS) divided by the degree of freedom (DF), F values are the ratio of the mean square to the true error, and *p* value is the probability of obtaining at least one statistic test. The outcomes of the ANOVA calculations for the toluene permeate flux and separation factor are shown in Table 3 and Table 4, respectively. On the other hand, the correlation coefficient (R^2^ values) for the separation factor and toluene permeation flux, which are both desired, were determined to be 99.37 and 99.07, respectively. This suggests that the two empirical models can account for around 99% of the data deviation [22].

Additionally, as can be seen from Table 5, the difference between R^2^ and predicted R^2^ is less than 0.20, which confirms the reliability of the data or model [49].

#### 3.4.2. Optimization of PV Process

**Figure 6:**
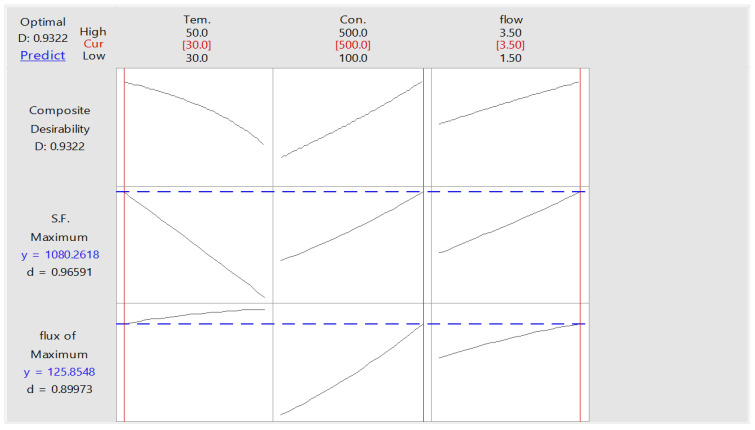
Optimization plot for toluene flux and *S.F.* for toluene–water solution.

One of the most popular approaches for the optimization of multiple response processes in the broad field of applied science and engineering is the desired function technique. With this approach, the individual desirability of several responses is combined into a single number with a range of 1.0 to 0. Since a value of 1 represents the ideal situation, values that are closer to 1 are preferred when describing the optimal operating conditions. On the other hand, if this value is near zero, it means that one or more responses are outside the desired range [50]. Thus, the desirability function of the current two responses (toluene flow and *S.F.*) was calculated using Minitab 18 software, combining the individual desirability into a single number, as shown in Table 6. Because it was predicted that these three variables would have the most impact on maximizing the toluene permeation flux and separation factor, this table lists the ideal operating conditions investigated (i.e., temperature, concentration, and flowrate). Thus, the results of the desirability function for the separation of toluene from the aqueous solution are displayed in Figure 6 and Table 6, respectively.

#### 3.4.3. Response Surface Plots of Multiple Effects

**Figure 7:**
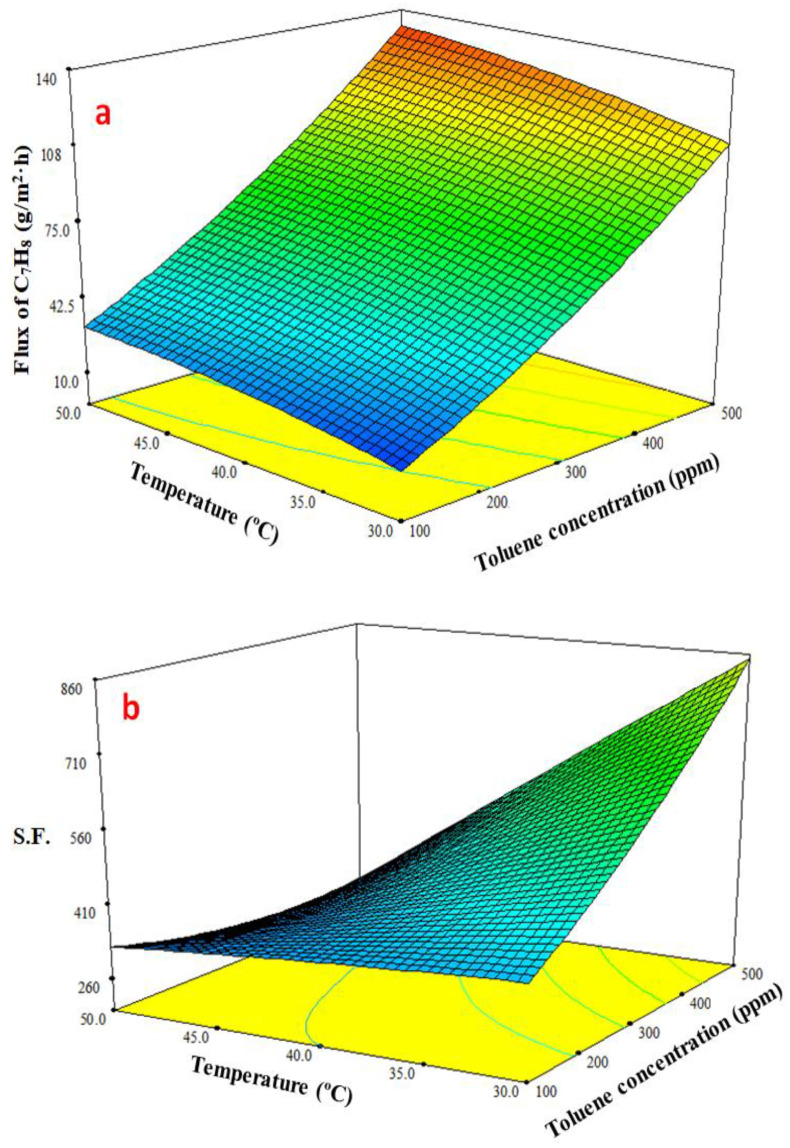
Effect the feed temperature and toluene concentration on (**a**) toluene flux and (**b**) separation factor (*S.F.*) at feed flowrate of 2.5 L/min.

The response surface plot in Figure 7a shows how the temperature and feed toluene concentration affect the toluene permeate flux. This graph demonstrates that the toluene permeation flux increased along with an increase in the feed temperature and toluene concentration. The toluene flux improved slightly when the feed temperature was raised from 30 to 50 °C, but the toluene concentration was more effective than the feed temperature and clearly increased the toluene permeation flux. Figure 7b response surface plot demonstrates how the initial toluene concentration in the feed and the feed temperature affect the separation factor. *S.F.* reduced with increasing the temperature as a result of the membrane swelling. Additionally, the rising temperature that promotes water diffusion reduces water clustering; consequently, toluene’s separation factor is reduced. In contrast, the *S.F.* increased as a result of a rise in toluene concentration [51].

**Figure 8:**
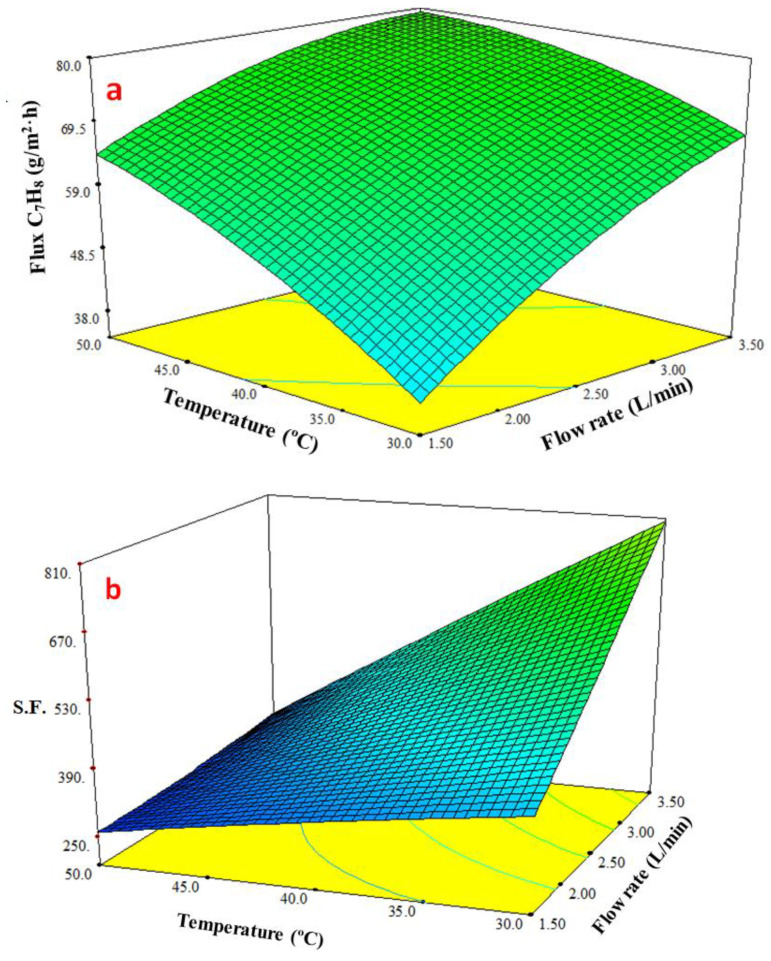
Effect the feed temperature and feed flow on (**a**) toluene flux and (**b**) separation factor (*S.F.*) at a concentration of toluene of 300 ppm.

In Figure 8, a response surface plot demonstrates how the feed flow and feed temperature affect the toluene flux. As the flexibility increased, the toluene permeate flow rose with rose the temperature. However, the lack of double bonds in PDMS makes it an extremely flexible polymer. This permits a high degree of rotation of the bonds, which makes it easier for penetrating species to diffuse through the free volume and causes a rise in membrane permeability with temperature [48]. Further, the boundary layer over the membrane surface was reduced as a result of the increasing feed flowrate, which led to a minor rise in the toluene permeate flux. After raising the feed temperature from 30 to 50 °C, the impact of the feed flowrate became clear. Figure 8b shows that the response surface plot demonstrates how the feed flow and temperature affect the *S.F.* for the toluene–water mixture. As was previously mentioned, the temperature had a negative impact on the *S.F.* because of membrane swelling. As a result, when the temperature was elevated, the *S.F.* reduced, and as a result, less toluene permeated through the membrane than the water. In contrast, an increase in feed flowrate increased the *S.F.* due to decreasing the boundary layer thickness. As a result, the mass transfer resistance of the boundary layer on the upstream membrane decreased, and the penetration flux of toluene increased.

**Figure 9:**
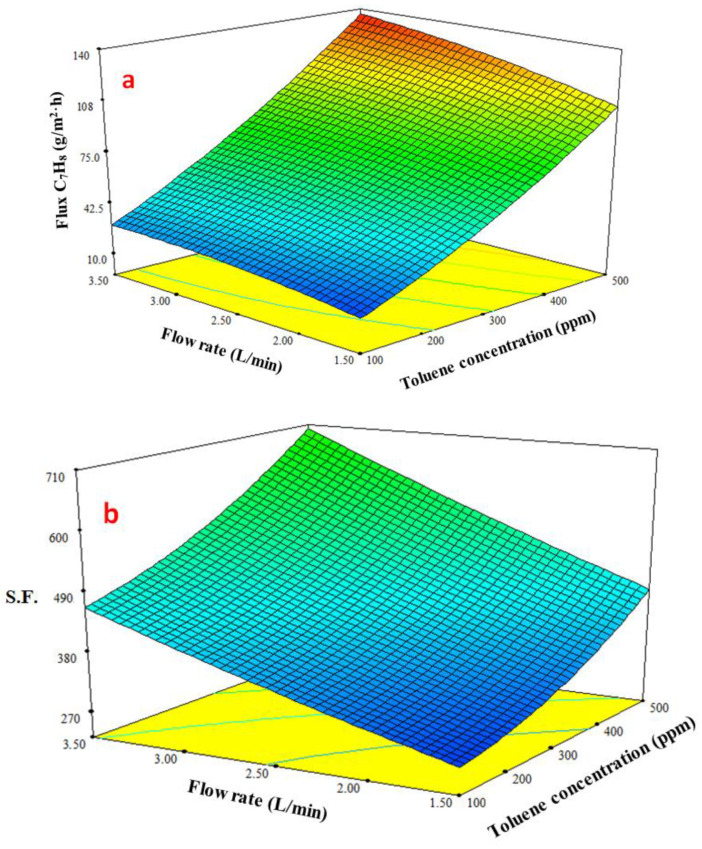
Effect the feed flow and concentration on (**a**) toluene flux and (**b**) separation factor (*S.F.*) at a temperature of 40 °C.

In Figure 9a, the response surface plot for the toluene permeation flux is displayed in three dimensions with the coupling effect of the interaction between feed concentration and feed flowrate. The toluene permeation flux increased more rapidly than the feed flowrate as the toluene content in the feed increased. The driving force (expressed by the concentration difference across the membrane) and the weakening of the boundary layer next to the membrane typically combine to increase the toluene penetration flow, where total permeation resistance is made up of boundary resistance and membrane resistance. The boundary layer’s thickness and boundary resistance both dropped as the flowrate increased. The response surface plot in Figure 9b shows how the feed flowrate and toluene content affect the *S.F.* Increasing toluene content in the feed raised the *S.F.* This outcome might be explained by the fact that the water molecules clustered as a result of hydrogen bonding, which decreased their permeability and diffusivity. Even at a low feed flowrate, the *S.F.* was enhanced by raising the toluene concentration.

## 4. Conclusions

Soluble toluene was recovered from water using PDMS membranes provided by DeltaMem AG (Switzerland) under various feed temperatures, toluene concentrations, and flowrate conditions. However, no research utilizing developed PDMS membranes to separate soluble toluene from water has been found in the literature. The current study concentrated on the PV process and a PDMS membrane’s ability to separate the soluble toluene component from an aqueous solution under various operating conditions. This study looked into how these three variables affected the permeate flux and separation factor. It was discovered that the permeate flux increased as the feed temperature, toluene concentration, and feed flowrate increased. However, the separation factor reduced as the feed temperature climbed but grew as the toluene concentration and feed flowrate increased. The combined impacts of these parameters on the permeate flux and separation factor were demonstrated using RSM methodology in Minitab 18 software. Analysis of variance and surface plots also offered a mathematical expression for calculating the flux and separation factor. The optimal response was established using composite desirability, with the following conditions: feed temperature of 30 °C, beginning toluene concentration of 500 ppm, and feed flowrate of 3.5 L/min. At these points, maximal output responses were both predicted and confirmed experimentally.

## Figures and Tables

**Table 1:** Experimental data points and response.

StdOrder	Temp. (°C)	Conc. (ppm)	Flowrate (L/min)	Flux (g/m^2^·h)	*S.F.*
5	30	100	3.5	20.3	575.0
9	30	300	2.5	53.0	560.0
14	40	300	3.5	80.0	523.0
12	40	500	2.5	122.0	528.0
15	40	300	2.5	70.0	447.0
19	40	300	2.5	70.0	447.0
6	50	100	3.5	30.5	351.0
18	40	300	2.5	70.0	447.0
3	30	500	1.5	87.0	629.0
7	30	500	3.5	127.0	1110.8
1	30	100	1.5	7.9	215.0
8	50	500	3.5	139.0	310.0
17	40	300	2.5	70.0	447.0
10	50	300	2.5	74.0	265.0
4	50	500	1.5	121.0	264.0
16	40	300	2.5	70.0	447.0
13	40	300	1.5	46.0	325.0
20	40	300	2.5	70.0	447.0
11	40	100	2.5	23.7	364.0

**Table 2:** The coefficients in Equations (4) and (5).

	A_0_	A_1_	A_2_	A_3_	A_4_	A_5_	A_6_	A_7_	A_8_	A_9_
$J_{T}$	−122.3	3.68	0.0356	34.1	−0.0273	16.5 × 10^−5^	−3.23	11.69 × 10^−4^	−0.361	0.02506
S.F.	−993	30.8	2.474	432.0	−0.046	72.1 × 10^−5^	6.9	−0.06480	−9.435	0.0792

**Table 3:** Analysis of variance for toluene permeate flux.

Source	DF	Adj SS	Adj MS	F-Value	*p*-Value
Model	9	26,443.9	2938.2	174.14	0.000
Linear	3	25,970.0	8656.7	513.05	0.000
Tem.	1	889.2	889.2	52.70	0.000
Con.	1	23,873.0	23,873.0	1414.85	0.000
flow	1	1207.8	1207.8	71.58	0.000
Square	3	124.7	41.6	2.46	0.122
Tem. × Tem.	1	20.5	20.5	1.22	0.296
Con. × Con.	1	120.5	120.5	7.14	0.023
Flow × flow	1	28.7	28.7	1.70	0.221
2-Way Interaction	3	349.1	116.4	6.90	0.008
Tem. × Con.	1	43.7	43.7	2.59	0.139
Tem. × flow	1	104.4	104.4	6.19	0.032
Con. × flow	1	201.0	201.0	11.91	0.006
Error	10	168.7	16.9		
Lack-of-Fit	5	168.7	33.7		
Pure Error	5	0.0	0.0		
Total	19	26,612.6			

DF = Degree of freedom, Adj SS = Sum of squares, Adj MS = Mean square.

**Table 4:** Analysis of variance for separation factor.

Source	DF	Adj SS	Adj MS	F-Value	*p*-Value
Model	9	697,438	77,493	118.98	0.000
Linear	3	485,146	161,715	248.29	0.000
Tem.	1	252,746	252,746	388.06	0.000
Con.	1	105,432	105,432	161.88	0.000
flow	1	126,968	126,968	194.94	0.000
Square	3	4697	1566	2.40	0.128
Tem. × Tem.	1	59	59	0.09	0.769
Con. × Con.	1	2290	2290	3.52	0.090
Flow × flow	1	129	129	0.20	0.666
2-Way Interaction	3	207,594	69,198	106.25	0.000
Tem. × Con.	1	134,369	134,369	206.31	0.000
Tem. × flow	1	71,215	71,215	109.34	0.000
Con. × flow	1	2010	2010	3.09	0.109
Error	10	6513	651		
Lack-of-Fit	5	6513	1303		
Pure Error	5	0	0		
Total	19	703,951			

**Table 5:** Model summary for toluene permeate flux and separation factor.

Parameters	Stand. Dev.	R^2^	R^2^(adj)	R^2^(pred)
Permeate flux	4.10769	99.37%	98.80%	94.04%
Separation factor	25.5207	99.07%	98.24%	90.53%

**Table 6:** Response optimization of toluene flux and *S.F.* for toluene–water solution.

Temp. (°C)	Conc. (ppm)	Flow (L/min)	*S.F.* Fit	Flux Fit (g/m^2^·h)	Composite Desirability
30	500	3.5	1080.26	125.855	0.932233

## Data Availability

The availability data of the current work is according to the experiment work and results are taken from the doctoral dissertation of the student, Salam H. Rasheed at the chemical engineering, university of technology, Iraq.
